# Color-Tunable Organic Light Emitting Diodes for Deep Blue Emission by Regulating the Optical Micro-Cavity

**DOI:** 10.3390/molecules25122867

**Published:** 2020-06-22

**Authors:** Jixin Jiang, Weiye Zheng, Junfei Chen, Zheng Xu, Dandan Song, Bo Qiao, Suling Zhao

**Affiliations:** 1 Key Laboratory of Luminescence and Optical Information, Ministry of Education, Beijing Jiaotong University, Beijing 100044, China; 19118027@bjtu.edu.cn (J.J.); 16118445@bjtu.edu.cn (W.Z.); 17118458@bjtu.edu.cn (J.C.); zhengxu@bjtu.edu.cn (Z.X.); ddsong@bjtu.edu.cn (D.S.); bqiao@bjtu.edu.cn (B.Q.); 2 Institute of Optoelectronics Technology, Beijing Jiaotong University, Beijing 100044, China

**Keywords:** micro-cavity, organic light emitting diodes, blue emission, spectral shift

## Abstract

Nowadays, most blue organic light emitting diodes (OLEDs) are fabricated by using sky-blue emitters which are more easily synthesized when compared with other deep blue emitters. Herein, we put forward a new idea of using an optical micro-cavity based on metal electrodes to regulate electroluminance (EL) spectra of sky-blue organic light emitting diodes to obtain a saturated deep blue emission with a narrowed full-width at half-maximum (FWHM). First, we simulate micro-cavity OLEDs and find that the transmission of the anode plays an important role in the forward emission. Meanwhile, the optical path of micro-cavity OLEDs as well as the phase shifting from electrodes influence the EL spectra and induce the extra intensity enhancement. The results show that when the resonant cavity optical path is regulated by changing the thickness of emitting layer (EML) from 25 nm to 75 nm in the micro-cavity, the EL peak of blue OLEDs has a redshift from 479 nm to 493 nm with FWHM shifting from 69.8 nm to 83.2 nm, when compared to the device without the micro-cavity, whose approximate EL peak and FWHM are 487 nm and 87 nm, respectively. However, the efficiency of electroluminescence decreases in micro-cavity OLEDs. We speculate that this is on account of the ohmic contact between ITO and Ag, the surface plasma effect and the rough morphology induced by Ag electrodes.

## 1. Introduction

As we all know, organic light-emitting diodes (OLEDs) have become the new generation of flat-panel display and solid-state light source for their excellent properties, which include solid-state active emitting-light and thinness, and they attract people’s attention for the rapid advancement and significant progress of OLEDs [1,2,3,4]. In particular, the property improvement of blue OLEDs is the key technique for achieving a better performance of OLEDs for their applications. Deep blue OLEDs are essential to flat-panel displays, mainly for reducing power consumption and saturated color performance. According to the National Television System Committee or the European Broadcasting Union (EBU) standard, deep blue color is usually defined as the blue electroluminescence (EL) emission with a Commission International de L’Eclairage (CIE) coordinate of y < 0.08 or y < 0.06 respectively [5,6]. To obtain saturated deep blue organic light emitting diodes, many studies are conducted in molecule design [7,8,9,10]. More recently, Li et al. designed a novel bipolar carbazole/phenanthroimidazole derivative named CzB-MOPPI, whose non-doped devices show the emission at 435 nm with a CIE of (0.16, 0.08), a maximum luminance of 6450 cd/m^2^, a maximum current efficiency (CE) of 3.34 cd/A and an external quantum efficiency (EQE) as high as 5.97% [11]. Kondo et al. reported a thermally activated delayed-fluorescence material that exhibits ultrapure blue emission, whose devices emit light at 469 nm with a full-width at half-maximum of 18 nm with an external quantum efficiency of 34.4% at the maximum and 26.0% at 1000 cd m^−2^ [12]. However, all these saturated deep-blue organic materials for full-color flat-panel displays have a deep energy level (HOMO) and a very wide band-gap [13,14]. It is quite difficult to design their molecular structure with a good carrier mobility and environment stability [15]. Among the various known blue organic materials, sky-blue emitters are considered to be the main molecule design [16,17,18]. Thus, we put forward a new idea to investigate a device structure to obtain the saturated deep blue emission based on sky-blue organic light-emitting diodes by introducing an optical cavity in devices.

As is known, an optical cavity can be formed in OLEDs by using high refractive index metallic electrodes [19,20,21,22,23] or DBR [24,25,26] composed of semi-conductors with a low and high ordered refractive index arrangement. Generally, metal electrodes are the preferred choices for their easy fabrication. Optical waves radiated from dipoles in the emitting layer (EML) reflect when they get to electrodes, which are regarded as parallel mirrors, and then following reflected light beams are subjected to the Fabry–Pérot interference. In other words, using electrodes can introduce the electromagnetic boundary conditions, and the photon density states can be altered strongly, with the states near the cavity resonance mode being enhanced while the others are weakened. This shows that the optical cavity in OLEDs enhances the emission solely at a particular color and suppresses others in the meantime, which is called micro-cavity. Li et al. reported that the micro-cavity structure markedly improves the efficiencies of green organic light-emitting diodes with a spectral peak intensity 4.3 times higher than that in a conventional device and a two-fold enhancement of the current efficiency [21], while the luminance enhancements of blue and red micro-cavity devices are small.

Here, we analyzed an optical resonant cavity, that is a micro-cavity in sky-blue OLEDs, on a theoretical basis to provide a convenient solution to saturated deep blue OLEDs. The results show that the emission of sky-blue OLEDs could be redistributed and that the spectra could be blue-shifted to the desired emission due to the multiple-beams interference and wide-angle interference by optimizing the device parameters. By introducing the micro-cavity effect into shy-blue OLEDs experimentally, we demonstrated that micro-cavity OLEDs possess a blue shift of the EL peak from 493 nm to 479 nm with a narrowing of the FWHM to 69.8 nm when compared to non-cavity devices with an approximate EL peak and FWHM of 487 nm and 87 nm, respectively, at the detriment of the luminance and current efficiency (CE).

## 2. Results and Discussion

### 2.1. Optical Properties of the Micro-Cavity in OLEDs

**Figure 1:**
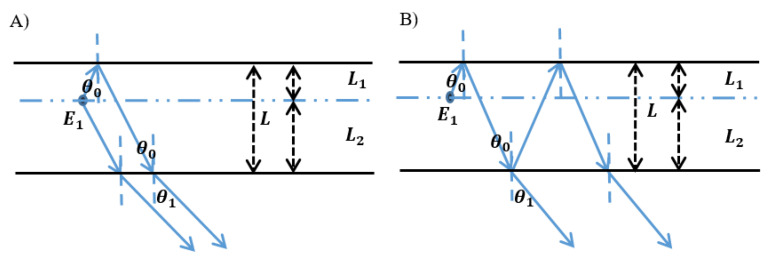
(**A**) Wide-angle interference and (**B**) multi-beam interference in micro-cavity bottom-emitting OLEDs. E_1_ represents the emission in the EML; θ_0_ and θ_1_ are incident angle and refraction angle on electrodes, respectively; L_1_, L_2_, L are the optical path from EML to two electrodes and between two electrodes, respectively.

It is reported that the micro-cavity is usually used in green OLEDs to enhance their intensity and efficiency by keeping the electroluminescence peak of emitters and resonance wavelength unchanged at the same time. Here, we use the micro-cavity to regulate the spectra of blue devices by changing the mode indexes. A multi-layer structure exists in bottom-emitting OLEDs (BOLEDs). Total reflection occurs on the internal surface of the cathode for its high reflectivity and thickness; as for the anode, semi-reflection occurs on it. This means that the emission of radiative dipoles reaches metal electrodes and produces a multi-beam interference with a wide-angle. The micro-cavity introduces the wide-angle interference and multi-beam interference into BOLED at the same time, as shown in Figure 1. Figure 1A shows wide-angle interference in micro-cavity bottom-emitting OLEDs and multi-beam interference is shown in Figure 1B. As shown in Figure 1, E_1_ represents the emission in the EML; θ_0_ and θ_1_ are incident angle and refraction angle on electrodes, respectively; L_1_, L_2_, L are the optical path from EML to two electrodes and between two electrodes, respectively.

**Figure 2:**
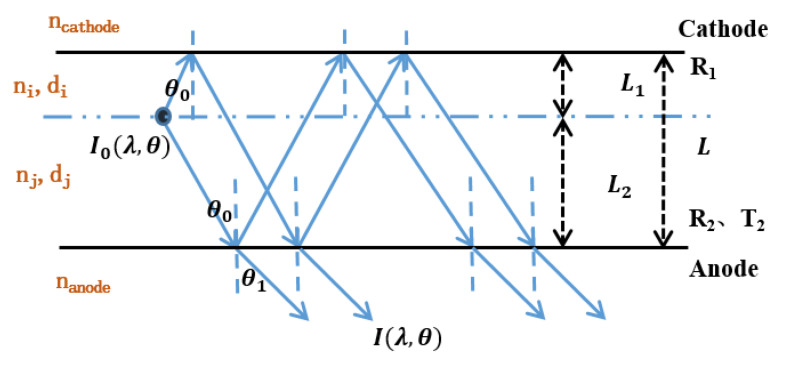
Micro-cavity OLEDs schematic.

In order to analyze the effect of this micro-cavity in BOLEDs, we implemented a theoretical simulation of its mechanism and found that the transmission of the anode plays an important role in the forward emission. Here, micro-cavity OLEDs can be simplified as a Fabry–Pérot resonator. In the resonator, a light source exists in the emitting layer between the parallel electrodes, as shown in Figure 2.

Assuming $I_{0}\left(\lambda ,\theta \right)$ as the emission of radiating dipoles in the emitting layer, after the wide-angle and multi-beam interferences inside the cavity, the light finally emits through the anode, which is semi-transparent. Then, the dividing amplitude is used to calculate the forward emission. The intensity of the forward emission I(λ,θ) from the micro-cavity has been calculated via Equations (1)–(4) [27,28,29,30]:

(1) $$I\left(\lambda ,\theta \right)=\frac{T_{2}\left(1+R_{1}+2\sqrt{R_{1}}\cos\left(\frac{4\pi L_{1}}{\lambda }-\varphi_{1}\right)\right)}{1+R_{1}R_{2}-2\sqrt{R_{2}R_{1}}\cos\left(\frac{4\pi L}{\lambda }-\varphi_{1}-\varphi_{2}\right)}I_{0}\left(\lambda ,\theta \right)\;$$

(2) $$L=L_{1}+L_{2}=\sum^{\;}n_{i}d_{i}+\sum^{\;}n_{j}d_{j}\;$$

(3) $$L_{1}=\sum^{\;}n_{i}d_{i}\;$$

(4) $$L_{2}=\sum^{\;}n_{j}d_{j}\;$$

(5) $$\varphi_{1},\varphi_{2},T_{2},R_{1},R_{2}=f\left(\lambda ,\theta \right)\;$$

Here, *R*_1_ and *R*_2_ are the reflective index of the cathode and anode, respectively, and *T*_2_ is the transmittance of the anode. *φ*_1_ and *φ*_2_ are the phase-shift angle from the cathode and anode, respectively, *n_j_* and *n_i_*, are the refractive index of each organic layer between the EML and anode or cathode, respectively, and, similarly, *d_j_* and *d_i_* are their thicknesses; n_anode_ and n_cathode_ are the refractive index of the anode and cathode, respectively. *L*_1_, *L*_2_ and *L* are the optical path from the radiative dipoles to the cathode, to the anode and between two metal electrodes, respectively.

In BOLEDs, the plane light is emitted from the emitting layer and incidents normal to electrodes. The reflectivity and transmissivity of metal electrodes can be calculated by the single-layer transfer matrix, which is obtained by Maxwell’s equations. Maxwell’s equations use four field quantities D, E, B, H and two sources J, ρ to demonstrate the variation of the electromagnetic field with the transfer matrix and thus to obtain a characteristic equation of the medium, which we can use for its optical constants, as in the following Equations (6)–(14):

(6) $$M_{m}=\left[\begin{array}{cc} A & B\\ C & D \end{array}\right]=\left[\begin{array}{cc} \cos\delta_{m} & \frac{i}{\eta_{m}}\sin\delta_{m}\\ i\eta_{m}\sin\delta_{m} & \cos\delta_{m} \end{array}\right]\;$$

(7) $$\eta_{m}=N_{m}y_{0}\;$$

(8) $$y_{0}=\sqrt{\frac{\varepsilon_{0}}{\mu_{0}}}\;$$

(9) $$\delta_{m}=\frac{2\pi }{\lambda }N_{m}d_{m}\cos\theta_{m}\;$$

(10) $$r=\frac{A\eta_{m}+B\eta_{m}\eta_{air}-C-D\eta_{air}}{A\eta_{m}+B\eta_{m}\eta_{air}+C+D\eta_{air}}=x+iy$$

(11) $$t=\frac{2\eta_{m}}{A\eta_{m}+B\eta_{m}\eta_{air}+C+D\eta_{air}}\;$$

(12) $$R_{m}=r\cdot r^{*}\;$$

(13) $$T_{m}=\frac{\eta_{air}}{\eta_{m}}t\cdot t^{*}\;$$

(14) $$\varphi_{rm}=\tan^{-1}\left[\frac{y}{x}\right]\;$$

By Equations (11)–(13), we can obtain the reflectivity R_m_, transmissivity T_m_ of the metallic electrode and its phase-shift *φ_rm_*. For the metallic electrode, M_m_ is the transfer matrix, and $\left[\begin{array}{c} B\\ C \end{array}\right]$ is its characteristic equation. r and t are the reflection coefficient and the transmission coefficient, respectively. *δ_m_* is the optical path of the electrode, and *η_m_*, *η_air_* are the optical admittance of medium and air. *y*_0_ is the optical admittance of free space, which can be considered as a unit. ε_0_, *μ*_0_ is the permittivity and magnetic conductivity of vacuum. *d_m_* is the thickness of the electrode, and *N_m_* is the complex refractive index of metal including the refractive index n and extinction coefficient κ and is calculated as in the following Equations (15) and (16):

(15) $$\varepsilon \left(\omega \right)=\varepsilon_{1}\left(\omega \right)+i\varepsilon_{2}\left(\omega \right)\;$$

(16) $$N_{m}=n+i\kappa =\sqrt{\varepsilon \left(\omega \right)}\;$$

The complex dielectric function (14) can describe the optical properties of the medium, and ε(ω) is usually called the dielectric constant, where (ω) and (ω) are referred from The Handbook on Optical Constants of Metals to calculate the N_m_ of the metal.

Figure 1 shows that the multi-beam interference and the wide-angle interference have a co-effect on the dipole emission. The enhancement generated at the same time from both two types of interference can be narrower and strengthen the forward emission. Equations (17) and (18) make clear the condition for the interference enhancement:

(17) $$\frac{4\pi L_{1}}{\lambda }-\varphi_{1}=2k\pi \;$$

(18) $$\frac{4\pi L}{\lambda }-\varphi_{1}-\varphi_{2}=2k^{\prime }\pi \;$$

$$k,k^{\prime }=0,1,2,3\ldots$$

Here, *k*, *k′* are the mode indexes corresponding to the resonance wavelengths. If the thickness of the organic layers is fixed as a constant, metallic electrodes play important parts in the micro-cavity effect for regulating the spectra of micro-cavity OLEDs precisely. Obviously, in a single resonant cavity whose cavity length is fixed, only one resonance wavelength can be emitted, and it is impossible to optimize the micro-cavity effects for different colors in a single resonant cavity.

In order to enhance the effect of Fabry–Pérot in a micro-cavity, a front semitransparent electrode with a high reflectivity and low absorption loss in the visible range is preferred, and Ag is mostly commonly used as the electrode to form a Fabry–Pérot cavity in OLEDs, due to its low extinction coefficient (k) value and simple fabrication steps. In addition, it is not easily oxidized in the air, and its high work function matches better with the HOMO level of the hole injection layer than other metals. In our experiments, the cathode material is aluminum (Al), and the optical simulation is carried out by using Matlab R2018a. According to Equations (6)–(14), we calculated the transfer matrixes of metallic electrodes and their phase shifts based on the Handbook of Optical Constants of Metals.

### 2.2. Design of the Micro-Cavity in Blue OLEDs

**Figure 3:**
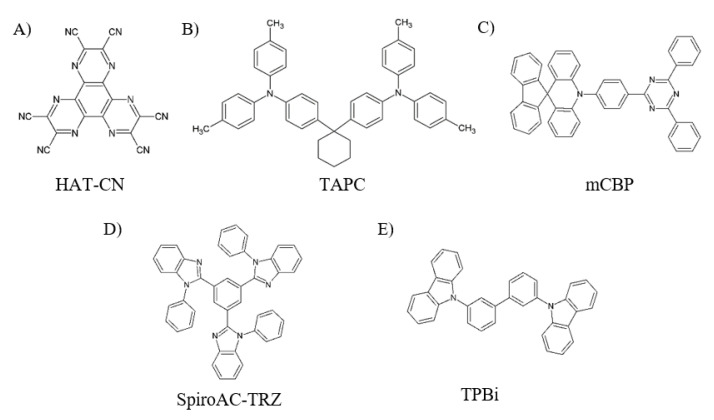
Molecule structure of organic materials: (**A**) HAT-CN, (**B**) TAPC, (**C**) mCBP, (**D**) SpiroAC-TRZ, (**E**) TPBi.

In order to obtain efficient blue OLEDs, 10-(4-(4,6-diphenyl-1,3,5-triazin-2-yl)phenyl)-10*H* -spiro [acridine-9,9’-fluorene] (SpiroAC-TRZ) has been chosen as the sky-blue emitter, 3,3-di(9*H*-carbazol-9-yl)biphenyl (mCBP) as the host, dipyrazino[2,3-f:2’,3’-h ]quinoxaline-2,3,6,7,10,11-hexacarbonitrile (HAT-CN) as the hole transporting layer (HTL), di-[4-(*N*,*N*-di-p-tolyl-amino)-phenyl]cyclohexane (TAPC) as the exciton blocking layer (EBL), 2,2′,2′’(1,3,5-benzenetriyl)tris-(1-phenyl–1*H*-benzimidazole) (TPBi) as the electron transporting layer (ETL) and lithium fluoride (LiF) as the electron injection layer (EIL) to match the energy level of each layer. Figure 3A–E shows the molecule structures of HAT-CN, TAPC, mCBP, SpiroAC-TRZ and TPBi, respectively.

**Figure 4:**
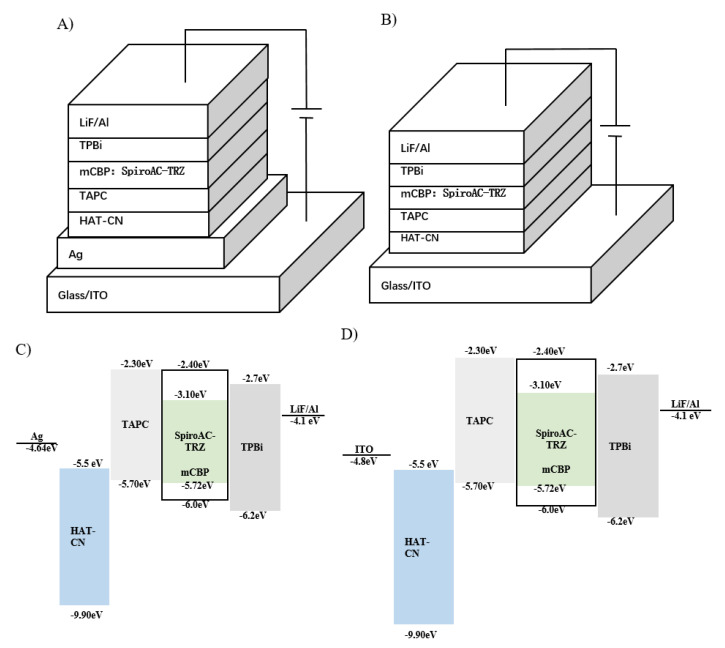
(**A**) Micro-cavity OLEDs device structure, (**B**) ITO-based OLEDs device structure, (**C**) Energy level of micro-cavity OLEDs, (**D**) Energy level of ITO-based OLEDs.

Figure 4 shows the schematic diagram of the micro-cavity OLEDs: A) with metallic electrodes and the control devices B) without the semi-transparent anode, corresponding to the energy level of C) and D), respectively. The refractive indices of these organic semiconducting materials have similar values ≈1.7–1.9 (n_org_ ≈1.7~1.9) in the visual light spectral region. Two kinds of OLEDs were simulated via Equations (1)–(5), taking the transfer matrix into consideration.

### 2.3. Theoretical Simulation of the Micro-Cavity in Blue OLEDs

**Figure 5:**
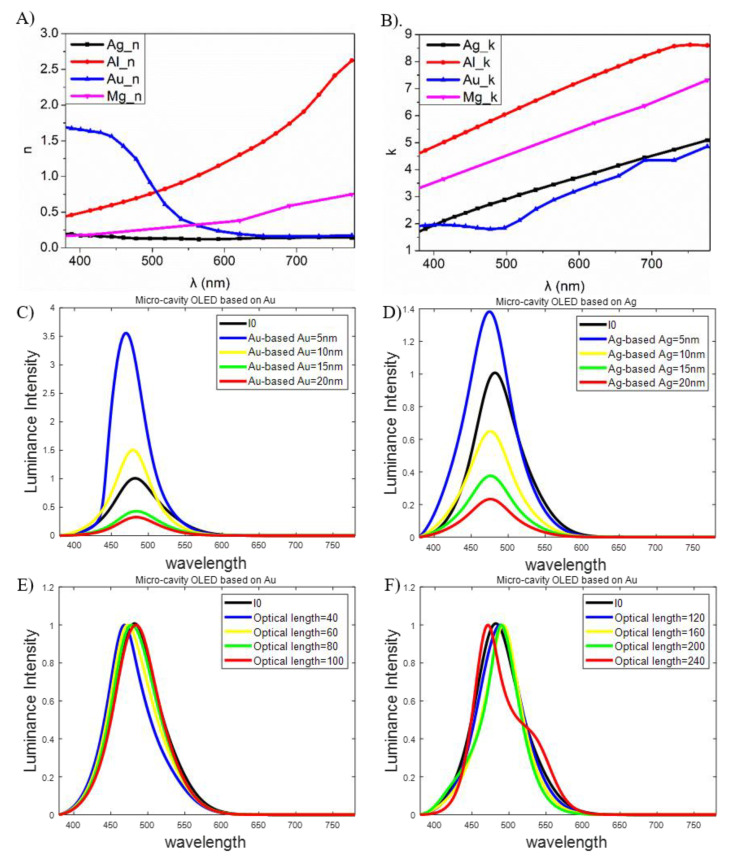
(**A**) Ordinary refractive indexes of different metals, (**B**) Extinction coefficients of different metals, (**C**) Simulated EL spectra of micro-cavity OLEDs based on Au with different thicknesses, (**D**) Simulated EL spectra of micro-cavity OLEDs based on Ag with different thicknesses, (**E**,**F**) Simulated EL spectra of micro-cavity OLEDs based on Au with different optical paths.

According to the Handbook of Optical Constants of Metals by Sadao Adachi, we fit the curve of refractive indexes of Ag, Al, Au and Mg as shown in Figure 5A,B, with n and κ as the ordinary refractive index and the extinction coefficient, respectively. The findings show that the absorption of Au is inferior to Ag, which means less optical loss. Thus, we simulate models of microcavities with Au and Ag layers with different thicknesses, respectively, where the optical length is set as 120 ($n_{\mathrm{org}}\approx 1.7\sim 1.9$), as shown in Figure 5C,D. $I_{0}$ is the emission of radiating dipoles in the emitting layer. It is apparent that Au-based electrodes can enhance the luminance intensity. However, as shown in Figure 5E,F, the Au layer is not suitable for the electrode because we need an ultrathin OLED for which the optical path between two electrodes is just 40 to make the peak wavelength be blue shifting; otherwise, in a conventional OLED where the optical path is 240, we will obtain the second peak, meaning approximately 170 nm-thick organic layers between electrodes. As a result, Ag is chosen as the electrode for the micro-cavity OLED.

**Figure 6:**
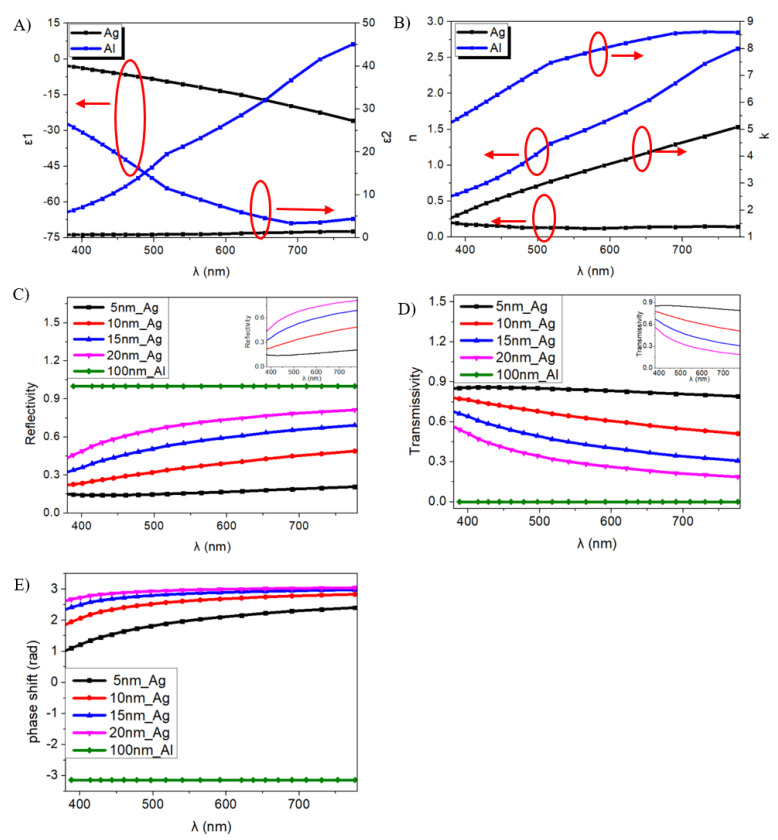
(**A**) Dielectric function, (**B**) refractive indexes, (**C**) simulated reflectivity, (**D**) simulated transmissivity of Ag and Al and (**E**) the simulated phase shift from Ag and Al.

Then, the simulated complex dielectric constants ε and complex refractive index *ñ* of Ag and Al are shown in Figure 6A,B, in which ε_1_ and ε_2_ is the real and the imaginary part of ε, and n and κ is the ordinary refractive index and the extinction coefficient, respectively.

According to Equations (6)–(13), Figure 6C–E shows the simulated reflectivity, transmissivity of the Ag layer with 5 nm-, 10 nm-, 15 nm- and 20 nm-thickness and of 100 nm-thick Al, as well as their phase shifts.

**Figure 7:**
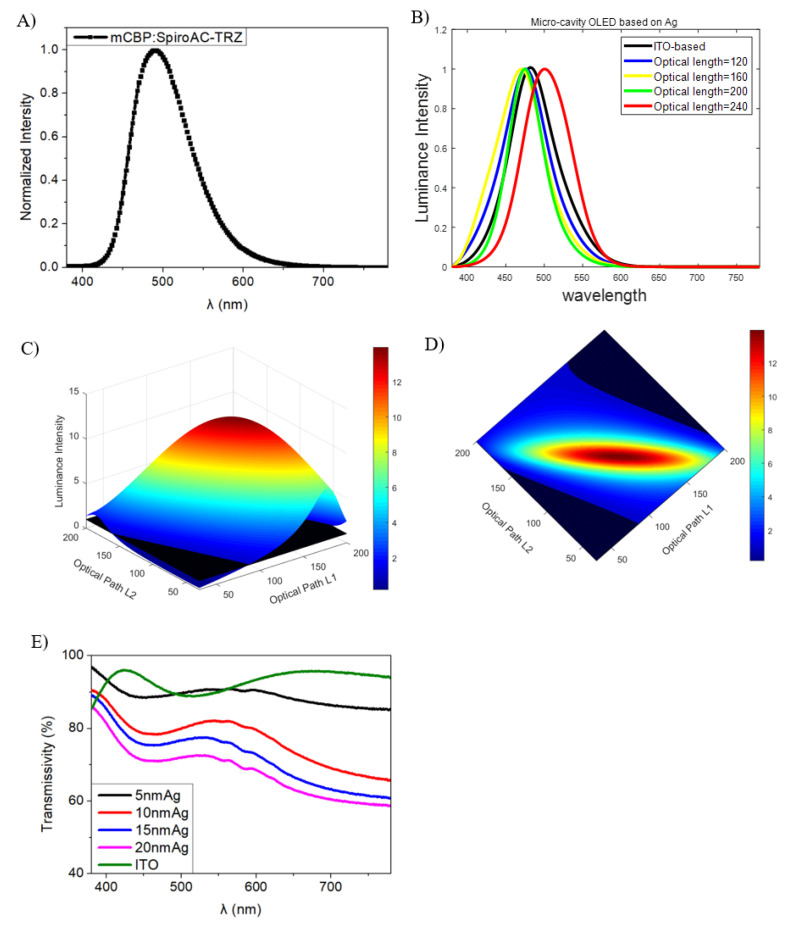
(**A**) PL spectra of mCBP doped with SpiroAC-TRZ, (**B**) Simulated EL spectra of micro-cavity OLEDs at different optical lengths, (**C**) Simulated luminance intensity of 480 nm in micro-cavity OLEDs with different optical paths L1 and L2, contrasted with ITO-based OLEDs (**D**) and its top view, and (**E**) the measured transmissivity of Ag (x nm, x = 5, 10, 15, 20)/ITO and ITO.

Obviously, thickness affects the optical constants of Ag and Al at different wavelengths. With an increasing thickness, the transmissivity of the Ag thin film decreases, while it has a perfect transmissivity at the range of 400–500 nm. Total reflection occurs for a 100 nm-thick Al layer, and its phase shift is unchanged during the visible region, which means that the phase shift stays the same when it reaches a certain thickness. Then, we measured the transmissivity of Ag with different thicknesses combined with ITO, as shown in Figure 7E, in order to make sure that the electroluminescence of OLEDs could be passed through. Apparently, there is a difference in transparence between Ag in its solid state by simulation and the Ag thin film in practice. It is found that the whole detected transmissivity decreases slightly with an increasing Ag thickness when combined with ITO in Figure 7E which is different from simulated transmissivity of Ag with different thickness in Figure 6D. It may be due to the light interference between the Ag and ITO layer.

Figure 7A describes the PL spectra with a PL peak of 482 nm of the emission layer of mCBP:SpiroAC-TRZ. Figure 7B shows the simulated EL spectra of the micro-cavity OLEDs with an Ag layer with different optical paths and of ITO-based OLEDs whose EL spectra were assumed to be similar to the PL spectra of mCBP:SpiroAC-TRZ due to the high transmissivity of ITO and the fact that it was normalized. With the highest transmissivity of all Ag layers, 5 nm-thick Ag enhances the luminance intensity because of its reflectivity contrast with ITO. By increasing the thickness of the Ag layer, the transmissivity decreases and the reflectivity rises to a similar degree, as shown in Figure 6C,D, while the forward emission of micro-cavity OLEDs with a 20 nm-thick Ag layer decreases to the minimum in simulated OLEDs. In addition, the phase shift also influences the intensity greatly. Figure 7C shows the simulated luminance intensity of 480 nm in micro-cavity OLEDs with L1 and L2 changing from 30 to 200, representing the optical path from EML to the cathode and anode, respectively. The surface and its top view in Figure 7C,D represent the intensity of micro-cavity OLEDs with a 10 nm-thick Ag layer, and the dark plane in this figure describes the intensity of ITO-based OLEDs. They show that the cavity length and the position of EML influence the spectra and intensity of the forward emission of OLEDs. There are two intersection lines between the dark plane and the surface of the emission of micro-cavity OLEDs shown in Figure 7D. The diagonal line from the coordinate point (50,50) to (200,200) also has two intersection points with two intersection lines. When comparing the x, y coordinate values of the intersection points, we can find that the optical path L1 has a stronger impact on the intensity than the optical path L2 does.

### 2.4. Deep Blue OLEDs Using Micro-Cavity

The transmission of the forward Ag layer recombined with ITO was detected, as shown in Figure 7F, to make sure that the electroluminescence of OLEDs could be passed through. The Ag thickness is 5 nm, 10 nm, 15 nm and 20 nm, respectively. In terms of forward emission, combined with Figure 5D, especially in the blue region, a better transmission and lower extinction coefficient are required. Meanwhile, the higher reflectivity and phase shift contribute to a constructive interference, which results in an extra enhanced intensity. However, the low wetting capability of the 5 nm-thick Ag layer causes un-continuous Ag islands. In view of the transparence and electric property, a 10 nm-thick Ag thin film is the appropriate choice. The devices used here are in a configuration of ITO/Ag (10 nm)/HAT-CN (20 nm)/TCTA (20 nm)/mCBP:SpiroAC-TRZ (wt.10%, xnm)/TPBi (20 nm)/LiF (1nm)/Al (100 nm). The control devices are: ITO/ HAT-CN (20 nm)/TCTA (20 nm)/mCBP:SpiroAC-TRZ (wt.10%, xnm)/TPBi (20 nm)/LiF (1 nm)/Al (100 nm).

**Figure 8:**
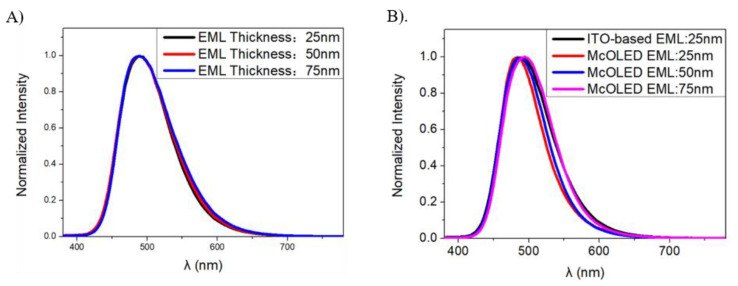
(**A**) EL Spectra of ITO-based OLEDs with EML with 25, 50 and 75 nm thicknesses, and (**B**) EL Spectra of micro-cavity OLEDs (McOLED) with EML with 25, 50 and 75 nm thicknesses.

As shown in Figure 8, the spectrum of devices is based on ITO with different thicknesses of EML from 25 nm to 50 nm; 75 nm changes little, and is almost same with an approximate EL peak and FWHM at 487 nm and 87 nm, respectively. Apparently, the EL peak of devices with the micro-cavity based on ITO/Ag (10 nm) blue-shifts to 479 nm, then turns to the red shift to 487 nm and 493 nm along with an increasing EML thickness. The shifting trend of EL peaks is consistent with the simulation results. It turns out that Ag thin film as anode leads to the blue-shifting of OLEDs’ spectrum, due to its reflectivity and obvious different refractive index when compared with organic layers, which brings about the phase shifting according to the simulation result shown in Figure 6E. The increased thickness of EML makes the spectrum red-shift because the optical paths L1 and L2 get longer. The reflected light contributes to the interference light, which is influenced and regulated by the joint effect of the phase shift and optical path, forming the EL spectrum. In addition to the peak blue shift, the FWHM of the spectrum of the micro-cavity OLED with an EML with a 25-nm thickness turns to 69.8 nm and is narrower than that of ITO-based OLEDs. When the spectrum red-shifts, its FWHM changes from 69.8 nm to 75.5 nm and 83.2 nm, respectively. All of the above show that the micro-cavity helps to suppress certain wavelengths and obtain a saturated deep blue by regulating the spectra.

**Figure 9:**
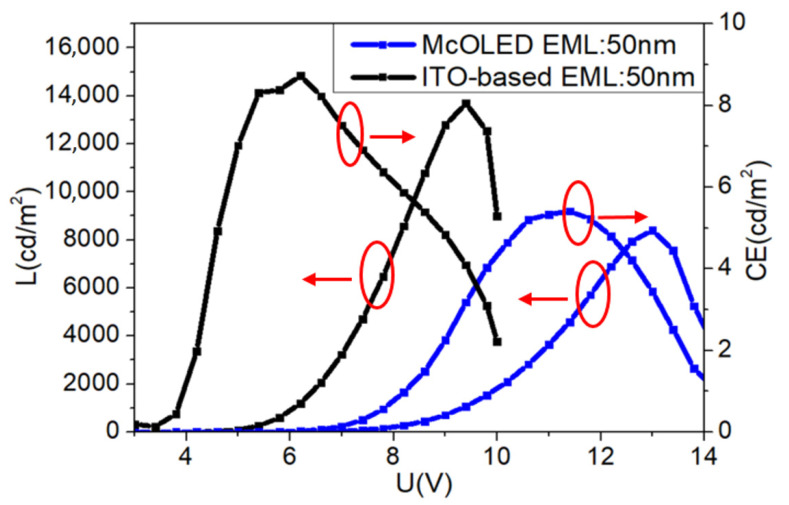
Current density-voltage-luminance of the ITO-based OLED and micro-cavity OLEDs.

As shown in Figure 9, compared to the control devices based on ITO with a 50-nm EML, the $V_{on}$ of the micro-cavity device increases and the luminance decreases, which is due to the extra resistance and low transmission resulting from the existence of the Ag thin film. We speculate that the contact between ITO and Ag results in the poor carrier injection, while the optical loss is caused by the surface plasma effect and defects or traps that Ag brings in for its ultrathin thickness. The 10 nm-thick Ag thin film can cause a point discharge because of its non-uniform morphology. Therefore, although the Ag thin film is easy to fabricate, the thinner micro-cavity OLEDs’ structure is easily broken down under a high current. Thus, the spectrum blue-shifting with the increased thickness of the Ag layer needs further investigation in such micro-cavity OLEDs in order to optimize their electroluminescence.

The electroluminescence device performances of ITO-based OLEDs and micro-cavity OLEDs are shown in Table 1. It shows that the micro-cavity structure obviously regulates the spectra of OLEDs, as the simulation shows. As for the intensity, EQE and CE, in order to establish a microcavity in the micro-cavity model in these OLEDs, the Ag electrode has the potential to improve the performance of OLEDs because of its optical properties and induced microcavity effect. However, there turns out to be a suppression of the intensity and current efficiency, and according to our experiments and analysis, this is mainly owing to defects and traps resulting from the rough morphology of the Ag film and SPP (surface plasmon polaritons) of Ag. In addition, the inferior adhesion of Ag to ITO is another problem related to the issue of a better performance. Besides these, owing to the low transparency of the 10 nm-thick Ag electrode, the luminance of the forward emission decreases with decreasing efficiencies. Thus, it is feasible to improve the performances of devices dramatically and change the spectra in the blue region with a 5 nm-thick Ag layer if we settle the problem of the point discharge.

## 3. Materials and Methods

### 3.1. Materials

10-(4-(4,6-diphenyl-1,3,5-triazin-2-yl)phenyl)-10*H*-spiro [acridine-9,9’-fluorene] (SpiroAC-TRZ) and 3,3’-Di(9*H*-carbazol-9-yl) biphenyl(m-CBP) were purchased from Taiwan Luminescence Technology Corp.

Dipyrazino[2,3-f:2’,3’-h ]quinoxaline-2,3,6,7,10,11-hexacarbonitrile (HAT-CN), di-[4-(*N*,*N* -di-p -tolyl-amino)-phenyl]cyclohexane (TAPC) and 1,3,5-tris(1-phenyl-1H-benzimidazol-2-yl)benzene (TPBi) were purchased from Xi’an Polymer Light Technology Corp.

### 3.2. Device Fabrication

All organic and inorganic materials used in this paper were commercially obtained without further purification. The devices used in our work were fabricated on patterned ITO-coated glass substrates with a sheet resistance of 15 Ω/sq, which were routinely cleaned in an ultrasonic bath with distilled water and ethyl alcohol. Then, the substrates were treated by O_2_ plasma under conditions of 1.3 × 10^−2^ Pa at 75 W for 3 min. All the layers were grown by thermal evaporation method at the base pressure of <5 × 10^−4^ Pa. The deposition rate was 0.1 nm/s for all organic layers and 0.01 nm/s for the LiF layer. Then, the Al cathode was deposited at a rate of 0.2 nm/s directly without opening the vacuum chamber. All the deposition rates and film thicknesses were monitored by a quartz crystal oscillator.

### 3.3. Device Measurements

The current density−voltage−luminance (J−V−L) characteristics and electroluminescence spectra of the OLED devices were measured simultaneously using a programmable source meter (Keithley model 2400) and a luminance meter/spectrometer (Photo Research PR655). All the measurements were conducted in ambient air. The transparencies of the ITO and Ag thin films were measured using an ultraviolet-visible spectrophotometer (UV-3100 spectrophotometer).

## 4. Conclusions

In summary, we make a theoretical analysis of micro-cavity OLEDs, and run a simulation of it. Our findings show that it is feasible to adopt a micro-cavity structure for regulating the visible spectra of OLEDs. We can get an expected blue shift by optimizing the optical path of devices and the phase shift from metallic electrodes. The simulation shows that transmission is essential for the forward emission of blue OLEDs, while reflectivity with the phase shift makes it possible to obtain a good performance. By performing experiments, we validated that using Fabry−Pérot interference in the micro-cavity can enhance the wavelengths we expected and suppress other wavelengths in order to regulate intrinsic EL peak blue shift precisely. By tuning the micro-cavity modes, the EL peak wavelength of blue OLEDs has a redshift from 479 nm to 493 nm, with an FWHM from 69.8 nm to 83.2 nm, compared to devices without a micro-cavity whose approximate EL peak wavelength and FWHM are 487 nm and 87 nm, respectively. However, in this paper, Ag-based micro-cavity OLEDs perform less well than DBR-based ones, for example in relation to a limited blue shift in the spectra and a suppression in intensity and current efficiency. As for mass production, Ag has the potential to be the alternative for ITO due to its good mechanical/electrical/optical properties and the fact that it is easy to fabricate. Thus, we plan to further investigate how to improve the performance of micro-cavity OLEDs based on Ag electrodes.

## Figures and Tables

**Table 1:** The electroluminescence device performances of ITO-based OLEDs and micro-cavity OLEDs.

Device	EML (nm)	Lmax (cd)	CE (cd/A)	Peak Wavelength (nm)	FMWH (nm)
ITO-based 1	25	5100	3.64	487	86
ITO-based 2	50	13,684	8.73	487	89
ITO-based 3	75	16,835	12	481	87
McOLED 1	25	1245.4	0.683	479	69.8
McOLED 2	50	8413.8	5.41	487	75.5
McOLED 3	75	8450	5.62	493	83.2
